# The Impact of Multidisciplinary Preoperative Optimization Program on Postoperative Outcomes Among Surgical Oncology Patients

**DOI:** 10.3390/jcm14217820

**Published:** 2025-11-04

**Authors:** Yasmin Safi, Mohammad S. Alyahya, Nihaya A. Al-sheyab, Mohammad Suliman, Mahmoud Al-Masri

**Affiliations:** 1 Department of Surgery, King Hussein Cancer Center, Amman 11941, Jordan; 2 Department of Health Management and Policy, Faculty of Medicine, Jordan University of Science and Technology, Irbid 22110, Jordan; msalyahya@just.edu.jo; 3 Allied Medical Sciences Department, Faculty of Applied Medical Sciences, Jordan University of Science and Technology, Irbid 22110, Jordan; nasheyab@just.edu.jo; 4 Community and Mental Health Department, School of Nursing, Al Al-Bayt University, Mafraq 25113, Jordan; mbarahemah@aabu.edu.jo; 5 Department of Surgery, School of Medicine, The University of Jordan, Amman 11942, Jordan

**Keywords:** preoperative, perioperative care, optimization, postoperative complications, outcomes, oncology, general surgery

## Abstract

**Background**: Preoperative optimization has emerged as a critical strategy in enhancing surgical outcomes, particularly for oncological patients. By addressing modifiable risk factors before surgery, healthcare providers aim to improve postoperative outcomes. The aim of this study was to evaluate the impact of a preoperative optimization program on postoperative outcomes and improvements in modifiable risk factors (anemia, malnutrition, smoking, and endocrine management) among oncology patients undergoing elective surgery. **Methods**: A retrospective pretest–posttest study was conducted including all oncology patients who underwent elective general surgery at King Hussein Cancer Center between January 2020 and December 2021. The preoperative optimization program was launched in May 2020 and fully implemented by December 2020. Program elements included anemia management, nutritional support, smoking cessation, and glycemic control. Patients were divided into pre-implementation and post-implementation cohorts, and outcomes were assessed at baseline, immediately preoperatively, and 30 days postoperatively. **Results**: The sample included 503 individuals, 53.9% had preoperative anemia, 15.5% had malnutrition, 40.6% were smokers, and 41.6% had uncontrolled DM. The optimized group demonstrated significant improvements in hemoglobin, albumin, and smoking cessation rates. In contrast, the control group showed worsening hemoglobin and albumin levels over the same period. Serious complications (Clavien–Dindo III–IV) were significantly more frequent in the control group (*p* = 0.006). The likelihood of postoperative complications among the control group was significantly higher than the optimized group (OR: 2.2, 95%CI: 1.5–3.2, *p* < 0.001). **Conclusions**: Implementation of a comprehensive preoperative optimization program significantly improved modifiable risk factors and reduced serious postoperative complications, highlighting its value for adoption in oncology surgical care.

## 1. Introduction

Cancer incidence is rapidly increasing worldwide, making timely and effective treatment essential to improve patient outcomes. According to the World Health Organization (WHO), cancer is the second leading cause of death globally, accounting for approximately 10 million deaths annually [1,2]. In Jordan, the burden of cancer is also rising, with 7594 of 10,006 newly diagnosed cases occurring among Jordanians. The most common cancer types in the country are breast (20.3%), colorectal (11.6%), lung (7.4%), lymphoma (6.9%), and leukemia (5.1%) [3].

Oncological treatment strategies vary according to cancer type, stage, and patient characteristics. Options include surgery with curative, palliative, or diagnostic intent, chemotherapy (neoadjuvant, adjuvant, or systemic), radiotherapy, targeted therapy, and immunotherapy. Among these, surgery remains the most frequently employed treatment modality. Optimal perioperative care, including careful preparation and recovery, is critical to maximize surgical outcomes [4,5].

Postoperative complications are among the most frequent adverse events in surgical oncology, with estimates suggesting that 7–15% of major surgeries result in complications ranging from minor infections to life-threatening events requiring further intervention. Postoperative mortality, typically assessed within 30 days of surgery, varies according to patient comorbidities, type of procedure, and other risk factors [6,7].

Modifiable preoperative risk factors—including smoking, malnutrition, anemia, elevated body mass index (BMI), and uncontrolled diabetes—are major contributors to postoperative complications. Addressing these factors before surgery has been shown to improve short-term outcomes, reduce surgical morbidity, shorten hospital stays, and enhance overall recovery. Preoperative evaluation serves as a baseline to assess functional health and guides decision-making for perioperative care [8,9,10]. Structured preoperative optimization programs, such as the American College of Surgeons’ Strong for Surgery (S4S) initiative, have been developed to engage both patients and surgeons in managing modifiable risk factors to improve surgical outcomes [11].

In this study, we evaluated the four domains of a preoperative optimization program—glycemic control, smoking, malnutrition, and anemia—and investigated their impact on postoperative complications among patients undergoing elective general oncology surgeries at King Hussein Cancer Center.

## 2. Materials and Methods

### 2.1. Study Design

A retrospective pretest–posttest (RPP) study design was utilized to evaluate the impact of a preoperative optimization program on postoperative outcomes. This design allows the assessment of interventions using existing data and provides a detailed understanding of the program’s influence over time. The RPP approach is widely used in longitudinal studies and is supported by recent empirical research [12,13].

### 2.2. Setting and Population

The study was conducted at the King Hussein Cancer Center (KHCC), a leading oncology treatment facility. Newly diagnosed adult oncology patients presenting to the surgical screening clinic between January 2020 and December 2021 were eligible if they underwent elective general surgery (breast, thyroid, upper gastrointestinal, or colorectal surgery).

Patients were divided into pre-implementation (control; January–December 2020) and post-implementation (Optimized; January–December 2021) cohorts based on the launch of the preoperative optimization program, which became fully operational in December 2020. Patients were observed at three time points: baseline (first encounter), immediate preoperative (1–2 days before surgery), and 30 days postoperatively.

Inclusion criteria: Adults (>18 years) undergoing elective, upfront, primary, or therapeutic surgery for breast, thyroid, upper gastrointestinal, or colorectal cancer.

Exclusion criteria: Pediatric patients, prior neoadjuvant therapy, optimized individuals, disease recurrence, urgent or non-primary diagnosis–related surgery, pregnant patients, and those with incomplete data regarding optimization status or outcomes.

A census sampling approach was used; all eligible patients meeting the criteria during the study period were included.

### 2.3. Preoperative Optimization Program

The preoperative optimization program at KHCC was modeled after the American College of Surgeons Strong for Surgery (S4S) initiative (American College of Surgeons, Chicago, IL, USA) [11]. S4S is a structured quality improvement program targeting modifiable perioperative risk factors, including nutrition, glycemic control, medication management, and smoking cessation. A Pareto analysis of general surgical patients with postoperative complications identified four priority domains for optimization:

1. Preoperative anemia—defined as hemoglobin < 13 mg/dL for men and <12 mg/dL for non-pregnant women. While the ideal goal was to achieve Hb ≥ 13 mg/dL in men or ≥12 mg/dL in women, full correction was not always feasible prior to surgery. Patients received appropriate interventions (e.g., iron supplementation, erythropoietin) to optimize Hb levels as much as possible before surgery, and their optimization status was documented accordingly.
2. Malnutrition—assessed using serum albumin (<3.5 mg/dL). Nutritional support (dietitian counseling, supplements) was provided to improve albumin levels before surgery. Full normalization to ≥3.5 mg/dL was the target, but when time constraints did not allow complete correction, patients were still considered partially optimized if interventions had been initiated and nutritional status improved as much as possible.
3. Smoking cessation—current smokers were referred to cessation counseling and support programs. Patients were considered optimized if they successfully abstained from smoking for at least two weeks prior to surgery.
4. Uncontrolled diabetes—defined as HbA1C > 7.5%. Medication adjustment or endocrinology consultation before surgery were considered optimized.

**Figure A1:**
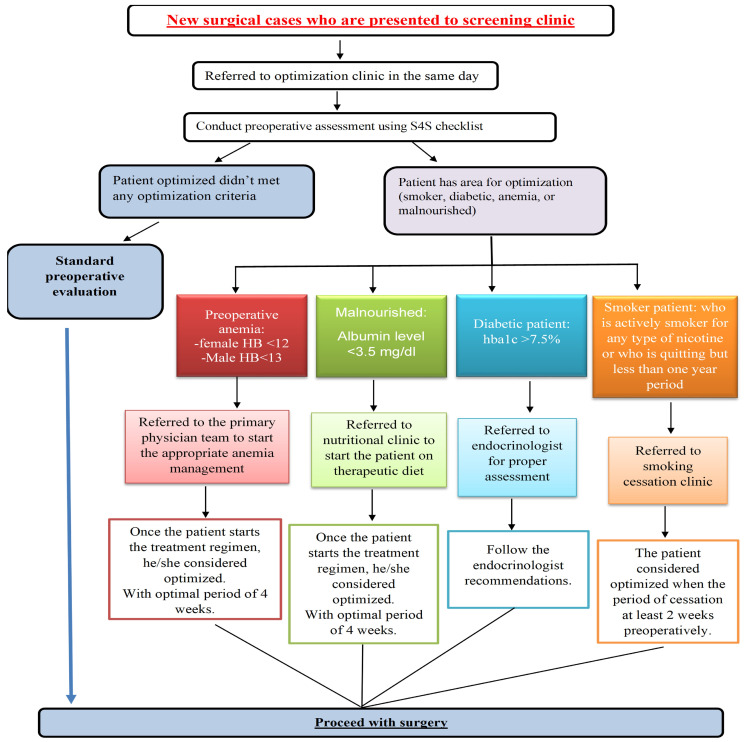
Workflow and Key Steps of the Preoperative Optimization Program.

The program included targeted interventions for each domain, with workflow integration into the surgical screening clinic (Figure A1). Patients’ optimization status was documented at baseline and immediately before surgery, allowing evaluation of program effectiveness in correcting modifiable risk factors.

### 2.4. Data Collection and Outcomes

Data were collected from electronic medical records and included patient demographics and baseline characteristics (age, sex, cancer type, and comorbidities), clinical variables (hemoglobin, serum albumin, HbA1C, and smoking status), and interventions received (anemia management, nutritional support, glycemic control, and smoking cessation counseling). The primary outcome of the study was the occurrence of postoperative complications within 30 days of surgery, classified according to the Clavien–Dindo grading system. Secondary outcomes included 30-day mortality, length of hospital stay (LOS), and readmission within 30 days. Additionally, the study assessed the effectiveness of the preoperative optimization program by measuring the proportion of patients achieving optimization in each of the four targeted domains (anemia, malnutrition, glycemic control, and smoking cessation) and evaluating the association between optimization status and postoperative outcomes. All variables and outcomes were recorded at baseline (first encounter), immediately preoperatively, and 30 days postoperatively to capture both the impact of the intervention and short-term surgical outcomes.

### 2.5. Statistical Analysis

Data analysis was performed using R software version 4.2.1 (R Foundation for Statistical Computing, Vienna, Austria). Descriptive statistics summarized patient demographics, baseline characteristics, clinical variables, and intervention uptake. Continuous variables were presented as means ± standard deviation or medians with interquartile ranges, while categorical variables were reported as frequencies and percentages. Inferential statistics were used to evaluate differences between pre-implementation (control) and post-implementation (optimized) groups. Paired-sample *t*-tests or Wilcoxon signed-rank tests were applied for within-group comparisons, and independent *t*-tests or chi-square tests were used for between-group analyses. The McNemar test was applied for paired categorical variables. Multivariable analyses were conducted to assess the effect of optimizing anemia, nutrition, glycemic control, and smoking cessation on postoperative morbidity, mortality, and blood transfusion rates. Binomial regression analysis was performed to estimate the odds ratios for postoperative complications between the optimized and control groups. Data manipulation and visualization were conducted using the dplyr package version 1.1.4 and ggplot2 package version 3.5.2. A *p*-value < 0.05 was considered statistically significant.

## 3. Results

Between January 2020 and December 2021, a total of 2872 new surgical cases were evaluated at the screening clinic. Of these, 2465 patients (85.8%) were classified as general oncology cases, while 235 patients (8.2%) presented with metastasis at diagnosis and 172 patients (6.0%) belonged to other surgical subspecialties. Among the general oncology cohort, 814 patients (33.0%) were successfully optimized according to the defined criteria (smoking status, hemoglobin, albumin, and HbA1C), whereas 1408 patients (57.1%) remained unoptimized and 243 patients (9.9%) had incomplete data regarding optimization status.

**Figure A2:**
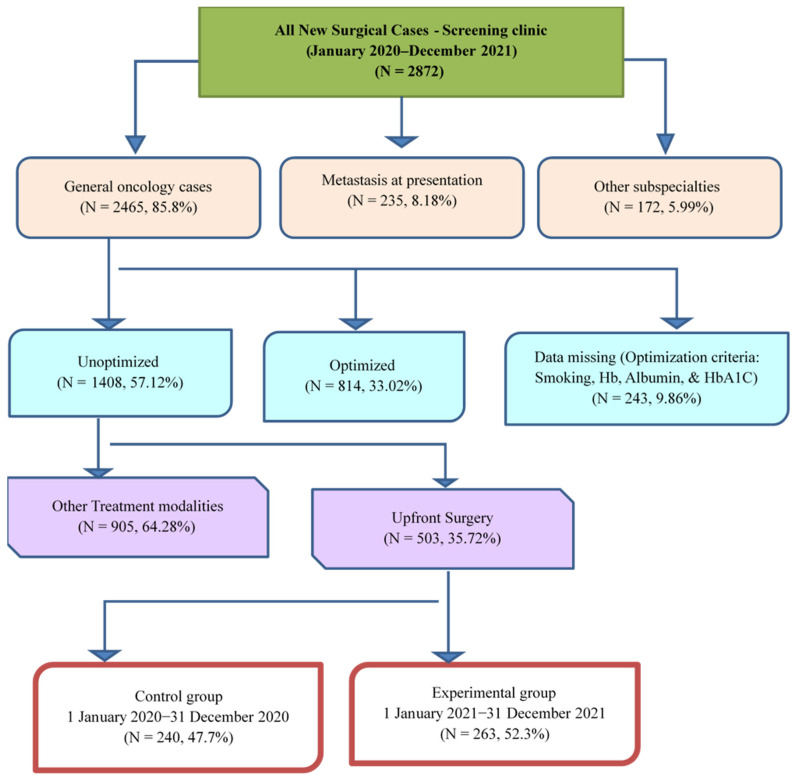
Patient Enrollment and Exclusion Flowchart (Final Sample Size).

Of the general oncology patients, 503 (35.7%) proceeded directly to upfront surgery, while 905 patients (64.3%) received alternative treatment modalities. Patients who underwent surgery were further stratified into two groups: the control group, consisting of 240 patients (47.7%) who underwent surgery between January and December 2020, and the optimized group, comprising 263 patients (52.3%) who underwent surgery between January and December 2021, following implementation of the preoperative optimization program (Figure A2).

### 3.1. Baseline Characteristics

The demographic and clinical characteristics of the surgical cohort are summarized in Table 1. The mean age of patients was comparable between the optimization group (54.18 ± 14.64 years) and the control group (54.34 ± 14.40 years, *p* = 0.905). The median age in both groups was 55 years. In terms of gender, females predominated in both cohorts (49.4% in the intervention group vs. 50.6% in the control group), though the difference did not reach statistical significance (*p* = 0.06). The distribution of cancer types varied significantly across the two groups (*p* < 0.001). Breast cancer was more prevalent in the control arm (58.6% vs. 41.4%), while colorectal cancer (59.5% vs. 40.5%) and gastrointestinal malignancies (69% vs. 31%) were more frequent among optimization program enrollees. Thyroid cancer cases were evenly distributed (47.6% vs. 52.4%). A significant difference was observed in disease staging (*p* = 0.041). Stage I disease was more common in the control group (56.1% vs. 43.9%), whereas Stage II (57.7% vs. 42.3%) and Stage III (53.9% vs. 46.1%) were more prevalent in the optimized group.

Comorbidity profiles were comparable, with 51.4% of the optimized group and 48.6% of the control group having one or more comorbid conditions (*p* = 0.553). Similarly, smoking status did not differ significantly between groups (51% vs. 49%, *p* = 0.628). Uncontrolled diabetes (HbA1c > 7.5%) was present in 52.1% of enrollees and 47.9% of controls (*p* = 0.94). Mean HbA1c levels were similar across groups (7.26 ± 2.33 vs. 7.36 ± 2.53, *p* = 0.627). Mean hemoglobin levels were slightly lower in the control group (11.9 ± 2.5 g/dL vs. 12.24 ± 2.3 g/dL), though the difference was not statistically significant (*p* = 0.076). Serum albumin levels were similar between groups (4.4 ± 0.95 g/dL vs. 4.6 ± 1.3 g/dL, *p* = 0.125), indicating no significant baseline differences in nutritional status (Table 1).

### 3.2. Program Adherence

Adherence to the preoperative optimization program demonstrated significant differences between the optimized and control groups across multiple domains. Nutritional referrals were documented exclusively in the optimized group, with all 32 patients (100%) receiving referrals compared to only 7 patients (15.2%) in the control group (*p* < 0.0001). Similarly, endocrine referrals were markedly higher in the optimized group, with all 112 patients (100%) being referred, whereas only 13 patients (12.6%) received referrals in the control group (*p* < 0.0001). Anemia management interventions were also more common in the optimized group, where 62.3% of patients received treatment, compared to 36.2% in the control group (*p* < 0.0001). Among the different anemia management strategies, intravenous (IV) iron therapy predominated in the optimized group (50.6%), whereas blood transfusions were more frequent in the control group (64.7%). Oral iron supplementation was utilized by both groups, though more commonly in the optimized cohort (38.3% vs. 29.4%). Smoking cessation interventions showed a similar trend, with all 104 smokers in the optimized group enrolled in a cessation program compared to only 21 patients (21%) in the control group (*p* < 0.0001). Importantly, smoking quit rates were significantly higher in the optimized arm (64.4%) than in the control arm (27%) (*p* < 0.0001). Furthermore, a longer duration of abstinence was more frequently observed in the optimized group. Specifically, 64.2% quit for 2–4 weeks, and 9 patients (100%) maintained abstinence for more than 4 weeks, compared to none in the control group (*p* = 0.035) (Table 2).

### 3.3. Preoperative Parameter Changes

Compared with the first encounter, patients enrolled in the optimization program demonstrated significant improvements in key preoperative parameters. Mean hemoglobin levels increased from 10.28 ± 1.8 g/dL to 10.65 ± 2.1 g/dL (*p* = 0.008), while serum albumin improved markedly from 2.74 ± 0.16 g/dL to 3.35 ± 0.32 g/dL (*p* < 0.0001). Smoking cessation was also notable, with the proportion of non-smokers rising from 60.5% to 85.9% (*p* < 0.0001).

In contrast, patients in the standard (non-enrolled) group showed a decline in hemoglobin levels (10.13 ± 1.9 g/dL to 9.28 ± 2.2 g/dL, *p* < 0.0001) and no meaningful change in albumin levels (2.8 ± 0.16 g/dL to 2.7 ± 0.26 g/dL, *p* = 0.38). Although smoking rates decreased modestly in the control group, the reduction was less pronounced compared to the optimized arm (41.7% to 30.4%, *p* < 0.0001) (Table 3).

### 3.4. Perioperative and Postoperative Outcomes

Overall perioperative characteristics were comparable between groups in terms of ASA classification and time to surgery. However, major procedures were more frequent in the optimized group (52.9% vs. 41.7%, *p* = 0.016). Mean length of hospital stay did not differ significantly between groups (4.1 ± 2.5 vs. 3.9 ± 2.8 days, *p* = 0.39).

Postoperative outcomes favored the optimized group. The rate of 30-day complications was significantly lower (25.5% vs. 39.6%, *p* = 0.001), with fewer patients experiencing higher-grade Clavien–Dindo complications (grades III–V). Blood transfusion and surgical site infection rates were also reduced in the optimized group (5.3% vs. 10.4%, *p* = 0.044; 3.0% vs. 8.8%, *p* = 0.006, respectively). Rates of seroma formation (7.2% vs. 15.4%, *p* = 0.004) and 30-day readmission (6.5% vs. 12.1%, *p* = 0.029) were significantly lower in the optimized arm. Differences in mortality, pneumonia, sepsis, AKI, and reoperation did not reach statistical significance, although a favorable trend was observed for the optimized group (Table 4).

### 3.5. Adjusted Odds Ratios for Postoperative Complications (Control vs. Optimized Group)

Multivariable logistic regression analysis was conducted to compare the odds of postoperative complications between participants in the control group and those in the optimized group, adjusting for disease stage, diagnosis, and severity of surgery. The control group had significantly higher odds of experiencing 30-day postoperative complications (OR = 2.19, 95% CI: 1.49–3.23, *p* < 0.001), 30-day mortality (OR = 3.55, 95% CI: 1.18–10.65, *p* = 0.024), 30-day readmissions (OR = 1.99, 95% CI: 1.05–3.82, *p* = 0.031), surgical site infections (OR = 3.35, 95% CI: 1.40–8.03, *p* = 0.007), anastomotic leaks (OR = 3.75, 95% CI: 1.42–9.86, *p* = 0.007), and perioperative blood transfusions within 72 h postoperatively (OR = 3.90, 95% CI: 1.89–8.19, *p* < 0.001).

Although the control group also demonstrated higher odds of 30-day reoperations, seroma formation, acute kidney injury (AKI), pneumonia, and sepsis, these differences did not reach statistical significance.

**Figure 1:**
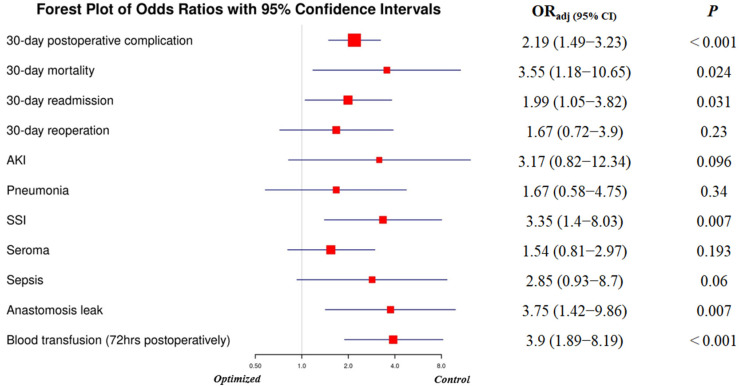
Adjusted odds ratios of postoperative complications in the control group. Abbreviations: AKI, acute kidney injury; SSI, surgical site infection; OR_adj_, adjusted odds ratio; CI, confidence interval.

Overall, these findings indicate that enrollment in the preoperative optimization program was associated with a significant reduction in several key postoperative complications, emphasizing the program’s effectiveness even after adjusting for disease stage, diagnosis, and surgical severity (Figure 1).

### 3.6. Subgroup Analysis of Postoperative Complications by Modifiable Risk Factors

**Figure 2:**
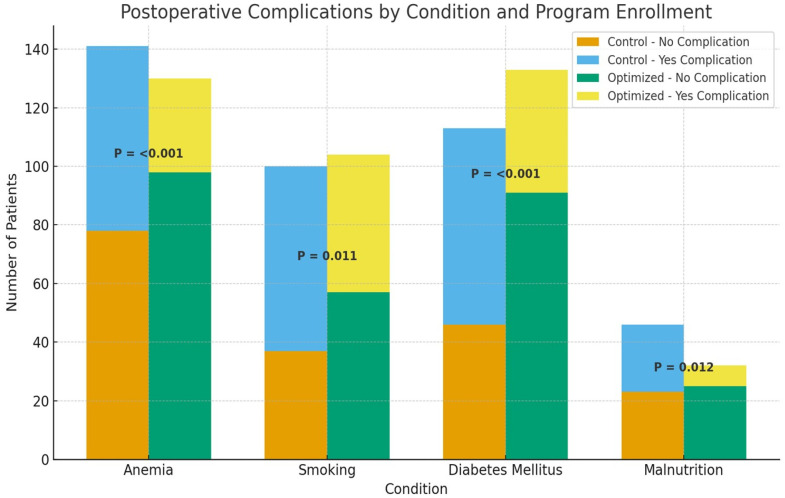
Stratified impact of preoperative optimization on postoperative complications across individual risk domains.

A stratified analysis of individual optimization domains showed that program enrollment significantly reduced postoperative complications among patients with anemia (24.6% vs. 44.7%, *p* < 0.001), uncontrolled diabetes (31.6% vs. 59.3%, *p* < 0.001), malnutrition (21.9% vs. 50.0%, *p* = 0.012), and smokers (45.2% vs. 63.0%, *p* = 0.011), indicating that optimization in each domain contributed to improved postoperative outcomes (Figure 2).

## 4. Discussion

This study demonstrated that implementation of a structured preoperative optimization program was associated with a significant reduction in postoperative complications among oncology patients undergoing elective surgery (27.4% vs. 41.8%, *p* < 0.001). Stratified analyses further showed that program enrollment markedly improved outcomes across individual domains, with complication rates reduced in patients with anemia (24.6% vs. 44.7%, *p* < 0.001), uncontrolled diabetes (31.6% vs. 59.3%, *p* < 0.001), malnutrition (21.9% vs. 50.0%, *p* = 0.012), and smoking (45.2% vs. 63.0%, *p* = 0.011).

Optimization programs are crucial interventions for improving postoperative outcomes by addressing modifiable health factors before surgery. By targeting risk factors such as anemia, malnutrition, and smoking, these programs not only reduce immediate postoperative complications but also support faster recovery, long-term health benefits, and potentially lower healthcare costs. The comprehensive nature of these programs creates a more favorable surgical environment, which is particularly important for high-risk populations such as oncology patients. Integrating optimization programs into standard surgical protocols represents a meaningful advancement in perioperative care, emphasizing the value of holistic patient management [14,15,16,17].

Consistent with our findings, MacMahon et al. (2022) [18] demonstrated that participation in a structured preoperative optimization program significantly reduced postoperative complications in total joint arthroplasty patients, including seromas, surgical site infections, and 30-day readmissions. Their study also established target thresholds for modifiable health indicators—HbA1c ≤ 7.5%, serum albumin ≥ 3.5 g/dL, and hemoglobin >12 g/dL for women or >13 g/dL for men—highlighting how correcting these factors preoperatively can decrease the risks of transfusions, infections, reoperations, and mortality. Our results closely align with these observations, further validating the effectiveness of comprehensive preoperative optimization programs in improving surgical outcomes [18].

Preoperative anemia is a well-established risk factor for adverse postoperative outcomes, highlighting the importance of effective anemia management strategies. In our study, anemic patients demonstrated higher complication rates, consistent with Musallam et al. (2011) [19], who reported significantly increased 30-day postoperative mortality across both mild and moderate-to-severe anemia. These findings support the value of comprehensive preoperative optimization programs that include targeted anemia management to reduce postoperative complications [19]. Yan et al. further identified independent risk factors for major morbidity after colorectal cancer surgery, including male sex, diabetes, open surgery, perioperative blood transfusions, and longer operative duration, which aligns with our observation of higher transfusion-related risk in the control group [20]. While some studies suggest that preoperative anemia may reflect overall poor health rather than being a direct cause of adverse outcomes, our results indicate that structured anemia optimization within a bundled preoperative program can effectively mitigate these risks and improve short-term surgical outcomes [20,21].

Smoking is a well-recognized modifiable risk factor associated with higher postoperative complication rates, including impaired wound healing, surgical site infections, and wound dehiscence. These complications can lead to unplanned reoperations, readmissions, and prolonged hospital stays [22,23,24,25,26,27]. In our study, patients who were current smokers and participated in the preoperative optimization program demonstrated a lower incidence of postoperative complications compared to non-optimized smokers. This likely reflects the program’s targeted interventions, including smoking cessation counseling and perioperative support, which mitigated the adverse effects of tobacco use [28,29]. Our findings are consistent with previous research, such as Yu-Hsuan Fan Chiang et al. (2023), which reported increased risks of wound disruption, infections, re-intubation, and in-hospital mortality among smokers [30].

Additionally, diabetes mellitus (DM) is a well-recognized independent risk factor for adverse postoperative outcomes, with poor glycemic control contributing to increased morbidity across surgical populations. In our cohort, patients with uncontrolled diabetes who participated in the preoperative optimization program experienced a substantial reduction in postoperative complications compared to controls. This improvement likely reflects the program’s targeted interventions, including individualized glycemic management, endocrinology consultations, and close perioperative monitoring, which collectively mitigated the risks associated with hyperglycemia. While studies by Wang et al. [31] and Wong et al. [32] demonstrated increased postoperative complications in patients with elevated HbA1c, our findings suggest that structured preoperative optimization can effectively modify this risk. The difference may also be attributed to our study population, which consisted exclusively of oncology patients—a high-risk group in whom intensive, bundled preoperative interventions may have a greater impact. These results underscore the value of proactive glycemic management within comprehensive preoperative optimization programs to improve surgical outcomes in high-risk patients.

A study conducted by the GlobalSurg Collaborative and the NIHR Global Health Unit on Global Surgery examined the impact of malnutrition on postoperative outcomes in patients undergoing elective general surgeries. The study involved 5709 patients from 381 hospitals across 75 countries and used the Global Leadership Initiative on Malnutrition criteria to define malnutrition. The findings revealed that severe malnutrition was present in 33.3% of patients, with a higher prevalence in low- and lower-middle-income countries (62.5%) and upper-middle-income countries (44.4%) compared to high-income countries. Severe malnutrition significantly increased the risk of 30-day mortality across all country income groups. Our study also found that patients presenting with malnutrition (hypoalbuminemia < 3.5 mg/dL) who did not participate in the preoperative optimization program experienced a significantly higher incidence of 30-day postoperative morbidity compared to those who were optimized (50.0% vs. 21.9%, *p* = 0.012). This underscores the effectiveness of targeted nutritional interventions within the program in reducing short-term complications and improving surgical outcomes for malnourished patients [33].

The study’s strength lies in its comprehensive evaluation of a structured preoperative optimization program, providing an in-depth understanding of its impact on multiple modifiable risk factors, including smoking cessation, anemia management, glucose control, and nutritional support. It employs rigorous statistical analysis and focuses on a high-risk population—oncology patients—highlighting its potential to enhance patient outcomes and overall surgical care quality.

However, the study was conducted at a single center, which may limit the generalizability of the findings to other surgical settings or non-oncology populations. The retrospective design may introduce inherent biases, and the short (30-day) follow-up period does not capture long-term outcomes, such as survival or quality of life. Additionally, subgroup sizes for certain risk factors were limited, and variability in program implementation could influence results. Patients were not randomized, as assignment to pre- and post-implementation cohorts was determined by the hospital-wide launch of the program. Although baseline characteristics and comorbidities were adjusted for in multivariable analyses, residual confounding factors such as temporal changes in surgical teams, hospital policies, or broader healthcare system dynamics cannot be fully excluded. Furthermore, while the study period overlapped with the COVID-19 pandemic, we accounted for its potential impact by including “time to surgery” in the analysis, which revealed no significant differences between cohorts; nevertheless, pandemic-related influences remain a potential source of bias. Other potential confounders, such as physical activity, psychosocial status, or socioeconomic factors, were not evaluated, as these were not systematically captured in our dataset. While the program was designed to target clinically modifiable risk factors that could be feasibly optimized in the preoperative window, the lack of broader patient-reported or social determinants of health is a limitation that may influence generalizability and outcome interpretation.

Future research should address these limitations by conducting prospective studies with extended follow-up and larger, more diverse patient populations, including multi-center implementation to improve generalizability. Standardized treatment protocols are also warranted to minimize variability in the delivery of optimization interventions. The next step is to evaluate the effectiveness of the preoperative optimization program in oncology patients undergoing neoadjuvant therapy followed by surgery, to assess its impact across the full perioperative care continuum.

## 5. Conclusions

Preoperative optimization programs significantly improve surgical outcomes by addressing modifiable risk factors such as smoking, anemia, hyperglycemia, and malnutrition. Implementation of these programs can reduce postoperative complications, enhance recovery, and promote more predictable, high-quality care. Healthcare organizations should consider adopting structured preoperative optimization protocols, supported by multidisciplinary teams, to maximize patient safety and outcomes. Ongoing evaluation and prospective studies are warranted to refine these programs and determine their long-term impact on surgical outcomes.

## Figures and Tables

**Table 1:** Baseline Characteristics of Surgical Oncology Patients by Study Group.

Variable	Level	Optimized Group 263 (52.3%)		Control Group 240 (47.7%)		*p* Value
Age (years)	Mean (±Std.)	54.18 (±14.64)		54.34 (±14.40)		0.905
	Median (Min–Max)	55 (22–87)		55 (19–85)		
Gender	Female	167	49.40%	173	50.60%	0.06
	Male	94	58.40%	67	41.60%	
Diagnosis Groups	Breast cancer	84	41.40%	119	58.60%	**<0.001**
	CRC	91	59.50%	62	40.50%	
	GI	58	69%	26	31%	
	Thyroid cancer	30	47.60%	33	52.40%	
Disease stage	I	65	43.90%	83	56.10%	**0.041**
	II	101	57.70%	74	42.30%	
	III	97	53.90%	83	46.10%	
Comorbidity	No	82	54.30%	69	45.70%	0.553
	Yes	181	51.40%	171	48.60%	
Smoking status	No	159	53.20%	140	46.80%	0.628
	Yes	104	51%	100	49%	
Uncontrolled DM (HbA1c > 7.5)	No	151	52.40%	137	47.60%	0.94
	Yes	112	52.10%	103	47.90%	
HbA1c	Mean (±Std.)	7.26 (±2.33)		7.36 (±2.53)		0.627
Hb level	Mean (±Std.)	12.24 (±2.3)		11.9 (±2.5)		0.076
Albumin	Mean (±Std.)	4.4 (±0.95)		4.6 (±1.3)		0.125

*p*-values were calculated using independent *t*-tests for continuous variables and chi-square tests for categorical variables. CRC: colorectal cancer; GI: gastrointestinal malignancies; DM: diabetes mellitus; HbA1c: glycated hemoglobin; Hb: hemoglobin. Values in **bold** indicate statistical significance at *p* < 0.05.

**Table 2:** Program Adherence: Optimization Interventions in the optimized and Control Groups.

Variable	Level	Optimized Group *N* (%)	Control Group *N* (%)	*p* Value
Nutritional referrals	No	0 (0%)	39 (84.1%)	<0.0001
	Yes	32 (100%)	7 (15.2%)	
Endocrine referrals	No	0 (0%)	90 (87.4%)	<0.0001
	Yes	112 (100%)	13 (12.6%)	
Anemia management	No	49 (37.3%)	90 (63.8%)	<0.0001
	Yes	81 (62.7%)	51 (36.2%)	
Types of anemia management	Blood transfusion	9 (11.1%)	33 (64.7%)	<0.0001
	IV	41 (50.6%)	3 (5.9%)	
	Oral	31 (38.3%)	15 (29.4%)	
Smoking cessation program	No	0 (0%)	79 (79.0%)	<0.0001
	Yes	104 (100%)	21 (21.0%)	
Quit smoking	No	37 (35.6%)	73 (73.0%)	<0.0001
	Yes	67 (64.4%)	27 (27.0%)	
Quitting duration (weeks)	<2 weeks	15 (83.3%)	3 (16.7%)	0.035
	2–4 weeks	43 (64.2%)	24 (35.8%)	
	>4 weeks	9 (100%)	0 (0%)	

*p*-values were calculated using chi-square or Fisher’s exact test, as appropriate. IV = intravenous.

**Table 3:** Changes in Preoperative Parameters between First Encounter and Preoperative Assessment in Optimized and Control Groups.

Variable	Level	Optimized Arm			Control Arm		
		First Encounter	Pre-Op	*t*-Test	First Encounter	Pre-Op	*t*-Test
Hb level (Anemic Group)	Mean (±SD)	10.28 (±1.8)	10.65 (±2.1)	2.67	10.13 (±1.9)	9.28 (±2.2)	−8.14
*p*-value		0.008			<0.0001		
Albumin level (Malnourished Group)	Mean (±SD)	2.74 (±0.16)	3.35 (±0.32)	6.95	2.8 (±0.16)	2.7 (±0.26)	−0.89
*p*-value		<0.0001			0.38		
Smoking status	Non-smoker	159 (60.5%)	226 (85.9%)		140 (58.3%)	167 (69.6%)	
	Smoker	104 (39.5%)	37 (14.1%)		100 (41.7%)	73 (30.4%)	
*p*-value		<0.0001			<0.0001		

*p*-values were calculated using paired *t*-test (for continuous variables: hemoglobin and albumin) and McNemar’s test (for categorical variables: smoking status).

**Table 4:** Perioperative and 30-Day Postoperative Outcomes: Optimized vs. Control Groups.

Variable	Level	Optimized Group (*N* = 263, 52.3%)	Control Group (*N* = 240, 47.7%)	*p*-Value
ASA	II	242 (92%)	219 (91.3%)	0.757
	III	21 (8%)	21 (8.8%)	
Severity of Surgery	Major	139 (52.9%)	100 (41.7%)	0.016
	Medium	98 (37.3%)	120 (50%)	
	Minor	26 (9.8%)	20 (8.3%)	
Time to surgery (days)	Mean ± SD	44.91 ± 19.8	43.3 ± 16.3	0.326
LOS (days)	Mean ± SD	4.1 ± 2.5	3.9 ± 2.8	0.39
30-Day Postoperative Complications	No	196 (74.5%)	145 (60.4%)	0.001
	Yes	67 (25.5%)	95 (39.6%)	
Clavien–Dindo Grade	0	191 (72.7%)	133 (55.4%)	0.006
	I	9 (3.4%)	10 (4.2%)	
	II	30 (11.4%)	43 (17.9%)	
	III	24 (9.1%)	38 (15.8%)	
	IV	4 (1.5%)	4 (1.6%)	
	V	5 (1.9%)	12 (5%)	
30-Day Mortality	No	258 (98.1%)	228 (95%)	0.081
	Yes	5 (1.9%)	12 (5%)	
Postoperative Blood Transfusion (≤72 h)	No	249 (94.7%)	215 (89.6%)	0.044
	Yes	14 (5.3%)	25 (10.4%)	
SSI	No	255 (97.0%)	219 (91.3%)	0.006
	Yes	8 (3.0%)	21 (8.8%)	
Pneumonia	No	257 (97.7%)	230 (95.8%)	0.229
	Yes	6 (2.3%)	10 (4.2%)	
Anastomosis Leak	No	256 (97.3%)	226 (94.2%)	0.076
	Yes	7 (2.7%)	14 (5.8%)	
Seroma	No	244 (92.8%)	203 (84.6%)	0.004
	Yes	19 (7.2%)	37 (15.4%)	
Sepsis	No	258 (98.1%)	229 (95.4%)	0.1
	Yes	5 (1.9%)	11 (4.6%)	
AKI	No	260 (98.9%)	232 (96.7%)	0.12
	Yes	3 (1.1%)	8 (3.3%)	
30-Day Readmission	No	246 (93.5%)	211 (87.9%)	0.029
	Yes	17 (6.5%)	29 (12.1%)	
30-Day Reoperation	No	254 (96.6%)	223 (92.9%)	0.06
	Yes	9 (3.4%)	17 (7.1%)	

*p*-values were calculated using independent *t*-test or Mann–Whitney test for continuous variables and χ^2^ or Fisher’s exact test for categorical variables. LOS = length of hospital stay; SSI = surgical site infection; AKI = acute kidney injury.

## Data Availability

The data presented in this study are available on reasonable request from the corresponding author. Data sharing is restricted due to privacy and ethical considerations, in accordance with Institutional Review Board approval and the waiver of informed consent.

## Abbreviations

The following abbreviations are used in this manuscript:

ACS	American College of Surgeon
AKI	Acute Kidney Injury
AL	Anastomosis leak
ASA	American Society of Anesthesiologists Score
AUC	Area Under Curve
CD	Clavien–Dindo Classification
CDC	Centers For Disease Prevention and Control
CRC	Colorectal Cancer
DM	Diabetes Mellitus
EMR	Electronic Medical Record
UGI	Upper Gastrointestinal
HB	Hemoglobin
HBA1C	Hba1c
NSQIP	National Surgical Quality Improvement Program
OP	Optimization program
OR	Odd Ratio
OR_adj_	Adjusted Odd Ration
POC	Postoperative Complications
QI	Quality Improvement
ROC	Receiver Operating Characteristic Curve
ROR	Return To Operation Room
RPP	Retrospective Pretest-Posttest
S4S	Strong for Surgery
SSI	Surgical Site Infection
WHO	World Health Organization

## References

[1] Siegel R.L., Miller K.D., Fuchs H.E., Jemal A. (2021). Cancer Statistics, 2021. CA A Cancer J. Clin..

[2] World Health Organization (WHO) Cancer. https://www.who.int/news-room/fact-sheets/detail/cancer.

[3] Ministry of Health Non-Communicable Diseases Directorate Jordan Cancer Registry (2019). Cancer Incidence in Jordan.

[4] Abbas Z., Rehman S., Shahzad H.N. (2018). An Overview of Cancer Treatment Modalities. Neoplasm.

[5] Leeds I.L., Canner J.K., Gani F., Meyers P.M., Haut E.R., Efron J.E., Johnston F.M. (2020). Increased Healthcare Utilization for Medical Comorbidities Prior to Surgery Improves Postoperative Outcomes. Ann. Surg..

[6] Martos-Benítez F.D., Gutiérrez-Noyola A., Echevarría-Víctores A. (2016). Postoperative Complications and Clinical Outcomes among Patients Undergoing Thoracic and Gastrointestinal Cancer Surgery: A Prospective Cohort Study. Rev. Bras. Ter. Intensiv..

[7] Dencker E.E., Bonde A., Troelsen A., Varadarajan K.M., Sillesen M. (2021). Postoperative Complications: An Observational Study of Trends in the United States from 2012 to 2018. BMC Surg..

[8] Pak H., Maghsoudi L.H., Soltanian A., Gholami F. (2020). Surgical Complications in Colorectal Cancer Patients. Ann. Med. Surg..

[9] Woodfield J.C., Jamil W., Sagar P.M. (2016). Incidence and Significance of Postoperative Complications Occurring between Discharge and 30 Days: A Prospective Cohort Study. J. Surg. Res..

[10] Aronson S., Murray S., Martin G., Blitz J., Crittenden T., Lipkin M.E., Mantyh C.R., Lagoo-Deenadayalan S.A., Flanagan E.M., Attarian D.E. (2020). Roadmap for Transforming Preoperative Assessment to Preoperative Optimization. Anesth. Analg..

[11] Varghese T.K., Chishimba S., Ma M., Ko C.Y., Flum D.R. (2019). The ACS Strong for Surgery Program: Changing Clinician and System Behavior to Optimize Health Before Surgery.

[12] Christie C.A., Fleischer D.N. (2010). Insight Into Evaluation Practice: A Content Analysis of Designs and Methods Used in Evaluation Studies Published in North American Evaluation-Focused Journals. Am. J. Eval..

[13] Young J. (2016). Retrospective Pre/Posttest Design and Response-Shift Bias in an Urban After-School Program for Teens: A Mixed Methods Study. Ph.D. Thesis.

[14] Robson M., Alexopoulou P. (2020). Pre-Optimisation of the Cancer Patient. Dig. Med. Res..

[15] Snowden C.P., Anderson H. (2012). Preoperative Optimization: Rationale and Process: Is It Economic Sense?. Curr. Opin. Anaesthesiol..

[16] Vine M., Joseph K., Gibson D., Lim B., Chua M., Siu A.H.Y., Dooreemeah D., Lee A., Cuomo R., Seth I. (2024). Innovative Approaches to Preoperative Care Including Feasibility, Efficacy, and Ethical Implications: A Narrative Review. AME Surg. J..

[17] Stokes S.M., Wakeam E., Antonoff M.B., Backhus L.M., Meguid R.A., Odell D., Varghese T.K. (2019). Optimizing Health before Elective Thoracic Surgery: Systematic Review of Modifiable Risk Factors and Opportunities for Health Services Research. J. Thorac. Dis..

[18] MacMahon A., Rao S.S., Chaudhry Y.P., Hasan S.A., Epstein J.A., Hegde V., Valaik D.J., Oni J.K., Sterling R.S., Khanuja H.S. (2022). Preoperative Patient Optimization in Total Joint Arthroplasty—The Paradigm Shift from Preoperative Clearance: A Narrative Review. HSS J..

[19] Musallam K.M., Tamim H.M., Richards T., Spahn D.R., Rosendaal F.R., Habbal A., Khreiss M., Dahdaleh F.S., Khavandi K., Sfeir P.M. (2011). Preoperative Anaemia and Postoperative Outcomes in Non-Cardiac Surgery: A Retrospective Cohort Study. Lancet.

[20] Yan T., Lei S., Zhou B., Huang Y., Li X., Zhang J., Huang Q., Zhang L. (2023). Association between Preoperative Anemia and Postoperative Short-Term Outcomes in Patients Undergoing Colorectal Cancer Surgery—A Propensity Score Matched Retrospective Cohort Study. BMC Anesthesiol..

[21] Srivastava V., Basu S., Shukla V.K. (2012). Seroma Formation after Breast Cancer Surgery: What We Have Learned in the Last Two Decades. J. Breast Cancer.

[22] Berriós-Torres S.I., Umscheid C.A., Bratzler D.W., Leas B., Stone E.C., Kelz R.R., Reinke C.E., Morgan S., Solomkin J.S., Mazuski J.E. (2017). Centers for Disease Control and Prevention Guideline for the Prevention of Surgical Site Infection, 2017. JAMA Surg..

[23] Goltsman D., Munabi N.C.O., Ascherman J.A. (2017). The Association between Smoking and Plastic Surgery Outcomes in 40,465 Patients: An Analysis of the American College of Surgeons National Surgical Quality Improvement Program Data Sets. Plast. Reconstr. Surg..

[24] Gräsbeck H.L., Reito A.R.P., Ekroos H.J., Aakko J.A., Hölsä O., Vasankari T.M. (2023). Smoking Is a Predictor of Complications in All Types of Surgery: A Machine Learning-Based Big Data Study. BJS Open.

[25] Nolan M.B., Martin D.P., Thompson R., Schroeder D.R., Hanson A.C., Warner D.O. (2017). Association between Smoking Status, Preoperative Exhaled Carbon Monoxide Levels, and Postoperative Surgical Site Infection in Patients Undergoing Elective Surgery. JAMA Surg..

[26] Sandy-Hodgetts K. (2019). Surgical Wound Complications: A 21st Century Problem?. J. Wound Care.

[27] Sørensen L.T. (2012). The Clinical Impact of Smoking and Smoking Cessation: A Systematic Rivew and Meta-Analysis. Arch. Surg..

[28] Yoshikawa R., Katada J. (2019). Effects of Active Smoking on Postoperative Outcomes in Hospitalised Patients Undergoing Elective Surgery: A Retrospective Analysis of an Administrative Claims Database in Japan. BMJ Open.

[29] Badiani S., Diab J., Woodford E., Natarajan P., Berney C.R. (2022). Impact of Preoperative Smoking on Patients Undergoing Right Hemicolectomies for Colon Cancer. Langenbeck’s Arch. Surg..

[30] Fan Chiang Y.H., Lee Y.W., Lam F., Liao C.C., Chang C.C., Lin C.S. (2023). Smoking Increases the Risk of Postoperative Wound Complications: A Propensity Score-Matched Cohort Study. Int. Wound J..

[31] Wang J., Chen K., Li X., Jin X., An P., Fang Y., Mu Y. (2019). Postoperative Adverse Events in Patients with Diabetes Undergoing Orthopedic and General Surgery. Medicine.

[32] Wong J.K.L., Ke Y., Ong Y.J., Li H., Wong T.H., Abdullah H.R. (2022). The Impact of Preoperative Glycated Hemoglobin (HbA1c) on Postoperative Complications after Elective Major Abdominal Surgery: A Meta-Analysis. Korean J. Anesthesiol..

[33] Riad A., Knight S.R., Ghosh D., Kingsley P.A., Lapitan M.C., Parreno-Sacdalan M.D., Sundar S., Qureshi A.U., Valparaiso A.P., Pius R. (2023). Impact of Malnutrition on Early Outcomes after Cancer Surgery: An International, Multicentre, Prospective Cohort Study. Lancet Glob. Health.

